# High rate of unplanned pregnancy in the context of integrated family planning and HIV care services in South Africa

**DOI:** 10.1186/s12913-018-2942-z

**Published:** 2018-02-27

**Authors:** Oladele Vincent Adeniyi, Anthony Idowu Ajayi, Mayowa Gabriel Moyaki, Daniel Ter Goon, Gordana Avramovic, John Lambert

**Affiliations:** 1 Department of Family Medicine and Rural Health, Walter Sisulu University, Cecilia Makiwane Hospital/East London Hospital Complex, East London, South Africa; 2 Department of Sociology, University of Fort Hare, 50, Church Street, East London, South Africa; 3 Department of Sociology, University of Ibadan, Ibadan, Nigeria; 4 Faculty of Health Sciences, University of Fort Hare, East London, South Africa; 5 University College Dublin/Mater Misericordiae University Hospital, Catherine McAuley Education & Research Centre, Dublin, Ireland

**Keywords:** Eastern cape, Family planning services, Unplanned pregnancy, Women living with HIV, South Africa

## Abstract

**Background:**

Integration of family planning services into HIV care was implemented in South Africa as a core strategy aimed at reducing unintended pregnancies among childbearing women living with HIV. However, it is unclear whether this strategy has made any significant impact at the population level. This paper describes the prevalence and correlates of self-reported unplanned pregnancy among HIV-infected parturient women attending three large maternity centres in the Eastern Cape, South Africa. We also compare unplanned pregnancy rates between HIV-infected parturient women already in care (who have benefitted from services’ integration) and newly diagnosed parturient women (who have not benefitted from services’ integration).

**Methods:**

Drawing from the baseline data of the East London Prospective Cohort Study (ELPCS), data of 594 parturient women living with HIV in the Eastern Cape were included. Chi-square statistics and binary logistics regression were employed to determine the correlates of unplanned pregnancy among the cohort.

**Results:**

The prevalence of unplanned pregnancy was 71% (*n* = 422) with a higher rate among parturient women newly diagnosed during the index pregnancy (87%). Unplanned pregnancy was significantly associated with younger age, single status, HIV diagnosis at booking, high parity and previous abortion. Women who reported unplanned pregnancy were more likely to book late and have lower CD4 counts. After adjusting for confounding variables, having one child and five to seven children (AOR = 2.2; CI = 1.3–3.1), age less than 21 years (AOR = 3.3; CI = 1.1–9.8), late booking after 27 weeks (AOR = 2.7; CI = 1.5–5.0), not married (AOR = 4.3; CI = 2.7–6.8) and HIV diagnosis at booking (AOR = 3.0; CI = 1.6–5.8) were the significant correlates of unplanned pregnancy in the cohort.

**Conclusion:**

Unplanned pregnancy remains high overall among parturient women living with HIV in the region, however, with significant reduction among those who were exposed to integrated services. The study confirms that integration of HIV care and family planning services is an important strategy to reduce unplanned pregnancy among women living with HIV. The study’s findings have significant implications for the elimination of mother-to-child transmission of HIV in South Africa. Innovative interventions are needed to further consolidate and maximise the benefit of the integration of family planning services with HIV care.

## Background

The majority of HIV-infected women are within the reproductive age group [1, 2]. Even though the availability of antiretroviral therapy has significantly reduced the risk of mother-to-child transmission of HIV and made prevention of mother to child transmission (PMTCT) possible, many women infected with HIV want to avoid pregnancy [3–7]. The reasons why many HIV-infected women prefer to avoid pregnancy are well documented. Among these reasons are the desire to stop bearing children, fear of premature death, financial constraints, having previously infected baby, and concerns about transmitting HIV to partners and children [6, 8, 9]. However, many of these women experience an unplanned pregnancy, which remains a major public health concern globally. In other words, the rate of unplanned pregnancies is alarmingly high in both developed and developing countries [6, 10–15].

South Africa, a country with a heavy burden of HIV [1], has made remarkable progress in the PMTCT of HIV. High rate of unplanned pregnancy in women infected with HIV remains a major concern [12, 16–18]. In 2012, a South African study showed that 62% of pregnancies among women with HIV were unplanned, with about half ending in abortion [12]. A high rate of unplanned pregnancy (68% to 85%) was also reported among HIV-infected women in the United States [14, 19]. Worryingly, a disproportionately higher rate of unplanned pregnancy was reported among adolescent women infected with HIV [15]. A high rate of unplanned pregnancy is commonly reported in the general population irrespective of women’s HIV status. A study has shown that there is no significant difference in the rate of unplanned pregnancy and contraceptive use between HIV sero-negative and sero-positive women [20]. In other words, sexual behaviours of HIV positive- and negative women are not dissimilar.

The question to ask is, “Why is unplanned pregnancy so high despite the availability of family planning services at the health facility level?”. As shown in the literature, contraceptive failure, incorrect and inconsistent condom use, and lack of knowledge of emergency contraception are among the reasons for high prevalence of unplanned pregnancy [21–23]. Absent father—a common phenomenon in South Africa context—has been linked to increased risk for early sexual activities and adolescent unplanned pregnancy [24]. In addition, younger age, never married status, experience of contraceptive failure, unemployment, early sexual debut, smoking, drug use, and lower education are among the factors associated with unplanned pregnancy [11, 13, 25–27]. The links between sexual risk taking and alcohol and substance use are well established and explain, in part, why unplanned pregnancy is prevalent globally [28–31].

The consequences of unplanned pregnancy are deleterious; among which are partner violence, depression, suicidal ideation, anxiety, and even death [13, 26, 27]. A Canadian study shows that unplanned pregnancy is associated with being unhappy [11]. The effect of unplanned pregnancy is not limited to women only; there is evidence that fathers facing unplanned pregnancies endure significantly more stress and often express a feeling of powerlessness in adapting to the arrival of a new infant [32]. Women with an unplanned pregnancy face an increased risk of abortion-related mortality, especially in countries with restrictive abortion laws [27]. Even in countries with permissive abortion laws, people often do not seek care due to abortion-related stigmas [33, 34], which are more prolific than HIV-related stigma [5].

Unplanned pregnancy is also an important indication of possible horizontal transmission of HIV. Some studies in the United States [13] and South Africa [11, 16] reveal that one-third of HIV-infected pregnant women partake in unprotected sex with a male partner with either negative or unknown status. Unplanned pregnancy, unprotected sex and transmission of HIV are intertwined and are often exacerbated through the transactional nature of sex [16, 35]. The complex nature of the link between transactional sex, poverty and unemployment makes it difficult to tackle the problem of unplanned pregnancy. Young adolescents are faced with increased risk of HIV infection and unplanned pregnancy as a result of intergenerational sex [17]. Vulnerable women, especially adolescents, tend to focus on tackling the more urgent risks in their lives: poverty, homelessness, and the frequent disruption of socio-economic support systems [36].

There is consensus among scholars that the integration of family planning services into HIV care, and PMTCT could potentially reduce the high rate of unplanned pregnancy, therefore, paving the way for a more cost-effective strategy to eliminate mother-to-child transmission of HIV [7, 15, 37–39]. A study has shown that averting unplanned pregnancy through the use of family planning messages and provision of contraception is a more cost-effective strategy than HIV counselling, testing and HAART provision in preventing perinatal HIV transmission [39]. However, a cluster randomised control trial study did not find any effect of service integration on the rate of unplanned pregnancy [38]. Nevertheless, service integration was associated with increased uptake of more effective contraceptive methods and condom use [38].

For over a decade, family planning services have been integrated into HIV services in South Africa with the aim of ensuring that all pregnancies are planned, and to prevent mother to child transmission of HIV. Women seeking reproductive health services are counselled on HIV and use of family planning. Also, contraceptives are freely given to women in all public health facilities. A good measure of the impact of service integration would be a measure of the rate of unplanned pregnancy in HIV-infected women in care. The reported rate of unplanned pregnancy would be important to track progress and address challenges. With a view to providing data for planning, monitoring and addressing the problem of unplanned pregnancy among women infected with HIV, this paper describes the prevalence and correlates of unplanned pregnancy in HIV-infected women attending maternal services in three large hospitals in the Eastern Cape, South Africa. Also, the study critically examines the impact of service integration by comparing the rate of unplanned pregnancy between women already engaged in HIV care and those who were newly diagnosed at the index pregnancy.

## Methods

### Study and sample design

The data analysed in this study was drawn from the baseline data of a prospective cohort study investigating PMTCT outcomes in Eastern Cape, South Africa. The data was collected in the three large hospitals serving over 1, 674,637 people of Buffalo and Amathole Districts in the Eastern Cape Province, South Africa. A total of 594 women infected with HIV were recruited. The sample size of 594 was estimated using the sample size calculator, at a confidence interval of ±4 and confidence level of 95%. All HIV-infected women attending childbirth care services during the period were recruited until the calculated sample size was reached. The study was conducted in December 2015 to May 2016. A full detail of the methodology was published elsewhere [40].

### HIV care and family planning services’ integration

HIV, Tuberculosis (TB), sexually transmitted diseases (STDs) and family planning services are integrated at all the primary health care centres in the country. This strategy of integration of services provides a platform for consolidation of care for patients in the study setting. Primary health care clinicians received training on the consolidated management of HIV, other STDs, TB, and family planning counselling and services including pregnancy test. This package of care is offered at every clinic visit in the study setting.

### Participants

Participants were previously- and newly diagnosed parturient women infected with HIV who delivered during their index pregnancies at the maternity units of Cecilia Makiwane, Frere and Bisho Hospitals. Participants were excluded if they were HIV negative.

### Measurement

Questions on a participant’s plan to conceive for the index pregnancy were elicited during an interview. Demographic and clinical characteristics were included in the questionnaire administered to respondents using an interviewer guided approach. Also, data on awareness of HIV serostatus and anti-retroviral therapy (ART) pre-conception were elicited. CD4 (cluster of differentiation 4) counts were extracted from the clinical records. Participants reported their age, marital status, type of residence, previous abortions and number of children including the index delivery. To measure the intent of pregnancy, participants were asked to provide a dichotomous response (Yes/No) to the question, “At the time you became pregnant, were you planning to become pregnant?”

We categorised the participants into two groups, namely, parturient women already in care (who have benefitted from services’ integration) and newly diagnosed parturient women (who have not benefitted from services’ integration). To examine the effect of services’ integration on the rate of unplanned pregnancy, we compared the proportion of women who reported an unplanned pregnancy between the two groups.

### Data analysis

Data were captured and analysed with the aid of Statistical Packages for Social Sciences (SPSS version 24). Frequency counts and percentages were estimated for all variables of interest. To examine correlates of unplanned pregnancy, chi-square statistics and binary logistics regression were employed. We examined the difference between the rate of unplanned pregnancy in previously diagnosed women (already in HIV and family planning care) and newly diagnosed women during pregnancy using chi-square statistics and binary logistics regression. A *p*-value less than 0.05 was considered to be statistically significant and the regression analysis was carried out at a 95% confidence level.

### Ethical clearance

The Walter Sisulu University Ethics Committee approved the study protocol (WSU/098/2014). Before the commencement of the study, permission was sought and granted by the Eastern Cape Department of Health and the Chief Executive Officers of the respective health facilities. Participants received verbal and written information detailing the purpose and process of the study. All participants provided a written, informed consent confirming their voluntary participation in the study. Confidentiality and privacy of medical information were maintained during the course of the study.

## Results

The mean age of the participants was 29.67 (SD = 6.40) years. The majority of the participants were single (65.5%), booked during the second trimester of their pregnancies (75.8%), and were aware of their HIV seropositive status before the index pregnancy (79.5%). About a quarter of the participants reported previous abortion(s) prior to the index pregnancy (Table 1). CD4 count and peripartum viral load were not available for 94 and 101 participants respectively.

**Table 1:** Demographic and clinical characteristics of participants

Variables	Frequency	Percentage
Age (years)		
Below 21	43	7.2
21–25	130	21.9
26–30	152	25.6
31–35	139	23.4
36–40	104	17.5
41–44	26	4.4
Marital status		
Married	116	19.5
Single	389	65.5
Co-habiting	73	12.3
Divorce/separated	16	2.7
Type of residence		
Rural	190	32.0
Semi-urban	262	44.1
Urban	142	23.9
^a^Gestational age at booking		
First	37	6.7
Second	417	75.8
Third	96	17.5
HIV serostatus at booking		
Negative	53	8.9
Positive	472	79.5
Unknown	69	11.6
^a^Parity		
1	194	32.7
2	204	34.4
3	117	19.7
4	56	9.4
5–7	22	3.7
^a^Previous abortions		
None	455	76.7
1	90	15.2
2	25	4.2
3	17	2.9
4	3	0.5
5	3	0.5
On HAART at booking		
Yes	316	65.8
No	164	34.2
CD4 counts at booking		
< 500	211	42.2
≥ 500	289	57.8

^a^missing data were excluded in the analysis

### Prevalence of unplanned pregnancy

**Fig. 1:**
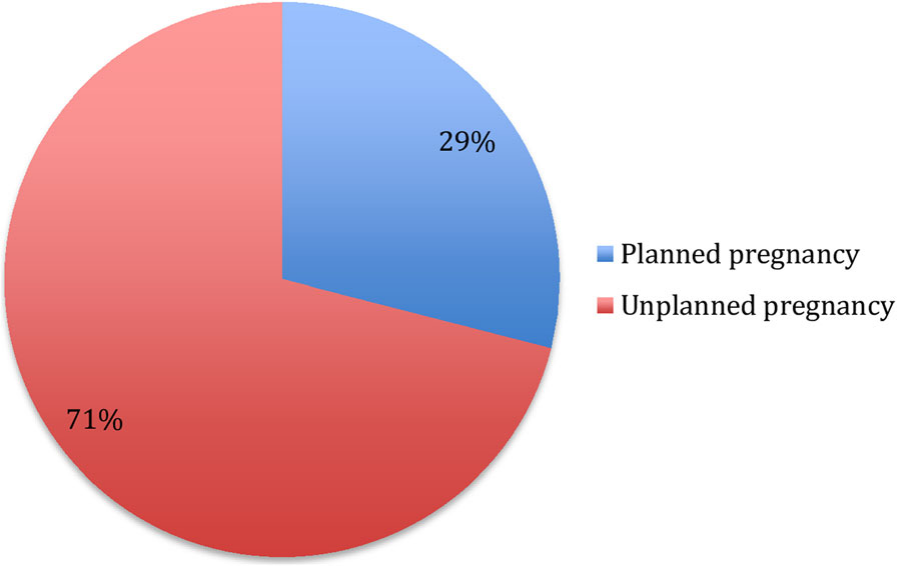
Rate of unplanned pregnancy in the cohort

The majority of the participants (*n* = 422; 71%) reported that the index pregnancy was unplanned (Fig. 1). The prevalence of unplanned pregnancy was highest among women who were single (81.7), aged below 21 years (90.7%), who had undergone three to five abortions (87.0%), who had five to seven children (90.9%) and who were HIV negative (86.8%) or unaware of their HIV serostatus at booking (87.0%) (Table 2).

**Table 2:** Demographic correlates of unplanned pregnancy

Variables	All *n* (%)	Planned pregnancy *n* (%)	Unplanned pregnancy *n* (%)	*p*-value
Age (years)				
Below 21	43 (7.2)	4 (9.3)	39 (90.7)	0.002
21–25	130 (21.9)	26 (20.0)	104 (80.0)	
26–30	152 (25.6)	49 (32.2)	103 (67.8)	
31–35	139 (23.4)	51 (36.7)	88 (63.3)	
36–40	104 (17.5)	34 (32.7)	70 (67.3)	
41–44	26 (4.4)	8 (30.8)	18 (69.2)	
Marital status				
Married	116 (19.5)	68 (58.6)	48 (41.4)	<0.001
Single	389 (65.5)	71 (18.3)	318 (81.7)	
Co-habiting	73 (12.3)	28 (38.4)	45 (61.6)	
Divorce/separated	16 (2.7)	5 (31.3)	11 (68.8)	
Type of residence				
Rural	190 (32.0)	51 (26.8)	139 (73.2)	0.165
Semi-urban	262 (44.1)	86 (32.8)	176 (67.2)	
Urban	142 (23.9)	35 (24.6)	107 (75.4)	
HIV serostatus at booking				
Negative	53 (8.9)	7 (13.2)	46 (86.8)	<0.001
Positive	472 (79.5)	156 (33.5)	316 (66.5)	
Unknown	69 (11.6)	9 (13.0)	60 (87.0)	
^a^Parity				
1	194 (32.7)	39 (20.1)	155 (79.9)	<0.001
2–4	377 (63.6)	131 (34.7)	246 (65.3)	
5–7	22 (3.7)	2 (9.1)	20 (90.9)	
^a^Previous abortions				
None	455 (76.7)	124 (27.3)	331 (72.7)	0.010
1	115 (19.4)	45 (39.1)	70 (60.9)	
3–5	23 (3.9)	3 (13.0)	20 (87.0)	

^a^missing data were excluded in the analysis

Unplanned pregnancy was significantly correlated with late booking (*p* = 0.01) and peri-partum CD4 immunosuppression (*p* = 0.028) (Table 3).

**Table 3:** Clinical correlates of unplanned pregnancy

Variables	All *n* (%)	Planned *n* (%)	Unplanned *n* (%)	*p*-value
^a^Gestational age at booking (trimester)				
First	37 (6.7)	10 (27.0)	27 (73.0)	0.01
Second	417 (75.8)	139 (33.3)	278 (66.7)	
Third	96 (17.5)	17 (17.7)	79 (82.3)	
^a^PP Viral Load				
Suppressed	275 (55.8)	84 (30.5)	191 (69.5)	0.618
Low Viraemia	128 (26.0)	33 (25.8)	95 (74.2)	
Virological failure	90 (18.3)	26 (28.9)	64 (71.1)	
^a^PP CD4				
Normal	188 (38.0)	63 (33.5)	125 (66.5)	0.028
Immunosuppression	307 (62.0)	77 (25.1)	230 (74.9)	

PP=Peripartum, ^a^missing data were excluded in the analysis using list-wise deletion

In the binary logistic regression, after adjusting for confounding variables (PP CD4 count and prior abortion), having one child or 5–7 children (AOR = 2.2; CI =1.3–3.1), unknown or negative HIV status at booking (AOR = 3.0; CI = 1.6–5.8), unmarried marital status (AOR =4.3; CI = 2.7–6.8), trimester above 27 weeks (AOR = 2.7; CI = 1.5–5.0) and age less than 21 years (AOR = 3.3; CI = 1.1–9.8) were the independent predictors of unplanned pregnancy (Table 4).

**Table 4:** Binary logistic regression showing predictors of unplanned pregnancy

Variables	Beta	Wald	AOR (CI)	*p*-value
Parity				
One child or 5–7 children	0.71	9.66	2.2 (1.3–3.1)	0.002
2–4 children (ref)			1	
HIV status at booking				
Negative or Unknown	1.10	10.93	3.0 (1.6–5.8)	0.001
Positive (ref)			1	
Marital status				
Not married	1.46	38.17	4.3 (2.7–6.8)	<0.001
Married				
Trimester at first visit				
Above 27 weeks	1.01	10.85	2.7 (1.5–5.0)	0.001
27 weeks and below (ref)			1	
Age				
Less than 21	1.19	4.61	3.3 (1.1–9.8)	0.032
21 and above (ref)			1	

*Ref*: reference, *AOR*: adjusted odd ratio, *CI*: confidence interval

Women who were not married were four times more likely to report unplanned pregnancy compared to women who were married. Likewise, women aged below 21 years were more than three times likely to report having an unplanned pregnancy compared to women who were 21 years or older. Women who delivered for the first time or those who had at least five children were twice more likely to report unplanned pregnancy compared to women who had two to four children..

In addition, women who booked late (greater than 27 weeks) were close to three times more likely to report unplanned pregnancy compared to women who booked within the first 27 weeks of the pregnancy. Also, women who were negative or unaware of their HIV positive status at booking were three times more likely to report unplanned pregnancy compared to women who already knew their status.

## Discussion

This study determines the prevalence of unplanned pregnancy and also, examines its correlates with a view to assessing the impact of the integrated services of family planning, HIV care and PMTCT. Our study shows that despite services integration, unplanned pregnancy is still very high and disproportionately high among women aged below 21 years, who have undergone three to five abortions, who have five to seven children, who are unaware of their HIV status at booking and single. The prevalence of unplanned pregnancy found in this study is higher than those reported by Schwartz et al., [12] in South Africa, Loutfy et al., [11] in Canada and Yanikkerem [27] in Turkey. However, a higher rate of unplanned pregnancy was reported by Sutton et al., [14]. The present study further shows that the majority of pregnancies in HIV-infected women are unplanned, despite family planning counselling and free access to contraceptives at HIV clinics in the region.

Plausible explanations for the high rate of unwanted pregnancies in our study include a high rate of unprotected sex irrespective of HIV serostatus among younger age group; discontinuation or irregular use of contraceptives; underutilization of emergency contraceptives and abortion services in the region [12, 18, 22, 23, 41]. In the South African communities, abortion-related stigma which is even worse than HIV-related stigma [5], could perhaps be among the reasons why many of these women decided to carry these pregnancies to term.

Our study shows that the rate of unplanned pregnancy is lower among women with a prior diagnosis of HIV in comparison with those who were diagnosed in the index pregnancy. This suggests a possible impact of services’ integration on reduction of unplanned pregnancy rate, contrary to Grossman et al., [38]. It is possible that the rate of unplanned pregnancy has declined in previously diagnosed women receiving care. There is evidence that the rate of unplanned pregnancy declined for the first time in the United States, which is a possible indication of the effectiveness of HIV care and family planning services’ integration [42].

The very high rate of unplanned pregnancy among adolescents and young women is not surprising, and is consistent with previous studies [15, 43]. Women below the age of 21 years are more than thrice likely to report an unplanned pregnancy compared to women aged 21 and above. The disproportionately high rate of unplanned pregnancy among adolescents and young women is perhaps due to the vulnerability of this age cohort. Older men often prey on vulnerable girls for transactional sex [17]. In Worth’s study [36], vulnerable women, especially adolescents, tend to focus on tackling the more urgent risks in their lives: poverty, homelessness and the frequent disruption of socio-economic support systems. Another plausible explanation is that adolescents and young women are more likely to engage in risky sexual behaviours including intergenerational sex [44–46].

Another important finding of this study is unmarried women being four times more likely to report an unplanned pregnancy compared to married women. This finding is consistent with previous studies [11, 47] and is not surprising, considering that conversations about pregnancy are more plausible in a marital relationship. The current study also reveals that late booking and peri-partum immunosuppression are associated with unplanned pregnancy. Low CD4 counts and late booking (after 27 weeks) could potentially have catastrophic implications for such women’s health. The finding stresses the need to ensure all pregnancies are planned. Innovative strategies to improve consistent use of contraception among women infected with HIV are needed in the study setting. Even though all the parturient women received peri-partum contraceptives prior to discharge, there is a need for continued engagement of these women in family planning services in order to prevent future unplanned pregnancies.

For over a decade, family planning services have been integrated into HIV services in South Africa as a core strategy to prevent mother-to-child transmission of HIV. Women seeking reproductive health services are counselled on HIV and use of family planning. Also, contraceptives are freely given to women in all public health facilities. The current study indicates a gap in the HIV services and reproductive health services’ integration. Our study also emphasises that while service integration is important, it is insufficient to address the problem of unplanned pregnancy in women infected with HIV. There is a need for constant monitoring of unplanned pregnancy rates in order to track progress and address challenges. Our study would serve as a baseline for future comparison and tracking of progress in the reduction of unplanned pregnancy rates in the region.

### Study limitations

It should be noted that quantitative method provides the extent of the burden of unplanned pregnancy in the study population; however, we could not gain an in-depth understanding of why pregnancies were unplanned among this cohort. Nonetheless, the large sample size drawn from multiple maternities in the Eastern Cape is a strength of this study.

## Conclusion

Unplanned pregnancy remains high overall among parturient women living with HIV in the region, however, with significant reduction among those who were exposed to integrated services. Our study confirms that integration of HIV care and family planning services is an important strategy to reduced unplanned pregnancy among women living with HIV. The study’s findings have significant implications for the elimination of mother-to-child transmission of HIV in South Africa. Innovative interventions are needed to further consolidate and maximise the benefits of integrating family planning services with HIV care.

## Abbreviations

AIDS: acquired immunodeficiency syndrome
CD4: cluster of differentiation 4
CI: confidence interval
HIV: human immunodeficiency virus
MTCT: mother-to-child transmission
PMTCT: prevention of mother to child transmission
